# Neural Network Deconvolution Method for Resolving Pathway-Level Progression of Tumor Clonal Expression Programs With Application to Breast Cancer Brain Metastases

**DOI:** 10.3389/fphys.2020.01055

**Published:** 2020-09-04

**Authors:** Yifeng Tao, Haoyun Lei, Adrian V. Lee, Jian Ma, Russell Schwartz

**Affiliations:** ^1^Computational Biology Department, School of Computer Science, Carnegie Mellon University, Pittsburgh, PA, United States; ^2^Joint Carnegie Mellon–University of Pittsburgh Ph.D. Program in Computational Biology, Pittsburgh, PA, United States; ^3^Department of Pharmacology and Chemical Biology, UPMC Hillman Cancer Center, Magee-Womens Research Institute, University of Pittsburgh, Pittsburgh, PA, United States; ^4^Department of Biological Sciences, Carnegie Mellon University, Pittsburgh, PA, United States

**Keywords:** breast cancer, brain metastases, phylogenetics, deconvolution, pathways, gene modules, transcriptome, matrix factorization

## Abstract

Metastasis is the primary mechanism by which cancer results in mortality and there are currently no reliable treatment options once it occurs, making the metastatic process a critical target for new diagnostics and therapeutics. Treating metastasis before it appears is challenging, however, in part because metastases may be quite distinct genomically from the primary tumors from which they presumably emerged. Phylogenetic studies of cancer development have suggested that changes in tumor genomics over stages of progression often result from shifts in the abundance of clonal cellular populations, as late stages of progression may derive from or select for clonal populations rare in the primary tumor. The present study develops computational methods to infer clonal heterogeneity and dynamics across progression stages via deconvolution and clonal phylogeny reconstruction of pathway-level expression signatures in order to reconstruct how these processes might influence average changes in genomic signatures over progression. We show, via application to a study of gene expression in a collection of matched breast primary tumor and metastatic samples, that the method can infer coarse-grained substructure and stromal infiltration across the metastatic transition. The results suggest that genomic changes observed in metastasis, such as gain of the *ErbB* signaling pathway, are likely caused by early events in clonal evolution followed by expansion of minor clonal populations in metastasis, a finding that may have translational implications for early detection or prevention of metastasis^1^.

## 1. Introduction

Metastatic disease is the primary mechanism by which cancer results in patient mortality (Chambers et al., 2002; Chaffer and Weinberg, 2011). By the time metastases have appeared, there are generally no viable treatment options (Guan, 2015). Successful treatment thus depends on treating not just the primary tumor but also the seeds of metastasis that may linger after a seemingly successful remission. Identifying successful treatment options for metastasis is problematic, however, since the genomics of primary and metastatic tumors may be quite different even in single patients and metastatic cell populations may be poorly responsive to therapies effective on the primary tumor. Studies of cell-to-cell variation in cancers have revealed often substantial clonal heterogeneity in single tumors, with clonal populations sometimes dramatically shifting across progression stages (Greaves and Maley, 2012). Phylogenetic studies of clonal populations have been inconclusive on the typical evolutionary relationships between primary and metastatic tumors (Schwartz and Schäffer, 2017). It remains a matter of debate whether changes in clonal composition occur primarily through ongoing clonal evolution, which results in novel clones with metastatic potential and resistance to therapy, or from selection on existing clonal heterogeneity already present at the time of first treatment (Ding et al., 2013; de Bruin et al., 2014). The degree to which either answer is true has important implications for prospects for early detection or prophylactic treatment of metastasis.

Brain metastases (BrMs) occur in around 10–30% of metastatic breast cancers cases (Lin et al., 2004). Although recent advances in the treatment of metastatic breast cancer have been able to achieve long-term overall survival, there are limited treatment options for BrMs and clinical prognoses are still disappointing (Witzel et al., 2016). Recent work examining transcriptomic changes between paired primary and BrM samples has demonstrated dramatic changes in expression programs over metastasis, including changes in tumor subtype with important implications for treatment options and prognosis (Priedigkeit et al., 2017; Vareslija et al., 2018). Some past research has sought to infer phylogenetic models to explain the development of brain metastases based on somatic genomic alterations (Brastianos et al., 2015; Körber et al., 2019). Such methods are challenged in drawing robust conclusions about recurrent progression processes, though, by the high heterogeneity within single tumors and across progression stages and patients. While single-cell methods are proving powerful for resolving such problems in other contexts (Qiu et al., 2011; Elyanow et al., 2020), such data is rarely available for studies of metastatic progression, which generally require working with samples archived years before metastases are discovered. Changes in the activity of particular genetic pathways or modules may provide a more robust measure of frequent genomic alterations across cancers.

In the present work, we develop a strategy for tumor phylogenetics to explore how changes in clonal composition, via both novel molecular evolution and shifts in population dynamics of tumor clones and associated stroma, influence changes in expression programs across such progression stages. Our methods make use of multi-site bulk transcriptomic data to profile changes evident in gene expression programs between clones and progression stages. We break from past work in this domain in that we seek to study not clones *per se*, as is typical in tumor phylogenetics (Eaton et al., 2018; Tao et al., 2019b), but what we dub “cell communities”: collections of clones or other stromal cell types that persist as a group with similar proportions across samples (section 2.4). We accomplish this via a novel transcriptomic deconvolution approach designed to make use of multiple samples both within and between patients (Schwartz and Shackney, 2010; Zare et al., 2014) while improving robustness to inter- and intra-tumor heterogeneity by integrating deconvolution with pathway-based analyses of expression variation (Park et al., 2009).

## 2. Materials and Methods

### 2.1. Overview

**Figure 1:**
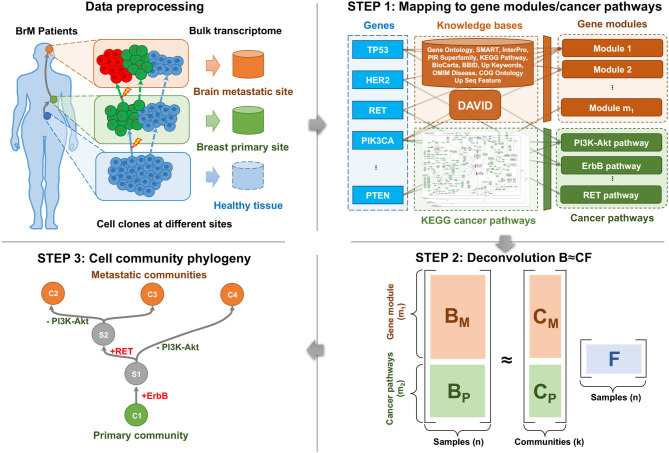
The pipeline of BrM phylogenetics using matched bulk transcriptome.

Cell populations evolve due to genomic perturbations that can result in changes in the activity of various functional pathways between clones. Our overall method for deriving coarse-grained portraits of cell community evolution at the pathway level is illustrated by Figure 1. After the preprocessing of transcriptome data (section 2.2), the overall workflow consists of three main steps: First, the bulk expression profiles are mapped into the gene module and pathway space using external knowledge bases to reduce redundancy, noise, and sparsity, and to provide markers of expression variation for the subsequent analysis (section 2.3). Second, a deconvolution step is implemented to resolve cell communities, i.e., coarse-grained mixtures of cell types presumed to represent an associated population of cancer clones and stromal cells, from the compressed pathway representation of samples (section 2.4). Third, phylogenies of these cell communities are built based on the deconvolved communities as well as inferred ancestral (Steiner) communities to reconstruct likely trajectories of evolutionary progression by which cell communities develop—through a combination of genetic mutations, expression changes, and changes in population distributions—as a tumor progresses from healthy tissue to primary and potentially metastatic tumor (section 2.5).

### 2.2. Transcriptome Data Preprocessing

We applied our methods to raw bulk RNA-Sequencing data of 44 matched primary breast and metastatic brain tumors from 22 patients (each patient gives two samples) (Priedigkeit et al., 2017; Vareslija et al., 2018), where six patients were from the Royal College of Surgeons (RCS) and sixteen patients from the University of Pittsburgh (Pitt). These data profiled the expression levels of ~60,000 transcripts. These can be represented in the format of a matrix, with rows corresponding to genes and columns to the samples (primary tumors or metastases). We removed the genes that are not expressed in any sample. We also considered only protein-coding genes in the present study. Approximately 20,000 genes remain after the filter. We conducted quantile normalization across samples using the geometric mean to remove possible artifacts (Amaratunga and Cabrera, 2001). The top 2.5% and bottom 2.5% of expressions were clipped to further reduce noise. Finally, we transformed the resulting bulk gene expression values into the log space and mapped those for each gene to the interval [0, 1] by a linear transformation. The resulting preprocessed transcriptome data were used as the input of Step 1 (section 2.3).

### 2.3. Mapping to Gene Modules and Cancer Pathways

The protein-coding gene expressions were mapped into both perturbed gene modules and cancer pathways, using the DAVID tool and external knowledge bases (Huang et al., 2009), as well as the cancer pathways in the KEGG database (Kanehisa and Goto, 2000). This step compresses the high dimensional data and provides markers of cancer-related biological processes (Figure 1, Step 1). Note that although both gene module and cancer pathway representations capture recurrent features of metastatic progression, they serve different purposes in our analysis. Gene modules are an essential part of deconvolution in the following steps because they provide the major variance within the data. Cancer pathways serve primarily as probes for *post-hoc* interpretation of the unmixed communities, but are biased relative to the gene module space by the focus only on genes with known relevance to cancer.

#### 2.3.1. Gene Modules

Functionally similar genes are usually affected by a common set of somatic alterations (Park et al., 2009) and therefore are co-expressed in the cells. These genes are believed to belong to the same “gene modules” (Desmedt et al., 2008; Tao et al., 2020). Inspired by the idea of gene modules, we fed a subset of 3,000 most informative genes out of the ~20,000 genes that have the largest variances into the DAVID tool for functional annotation clustering using several databases (Huang et al., 2009). DAVID maps each gene to one or more modules. We did not force the genes to be mapped into disjunct modules because a gene may be involved in several biological functions and therefore more than one gene module. We removed gene modules that were not enriched (fold enrichment < 1.0) and kept the remaining *m*_1_ = 109 modules (and the corresponding annotated functions), where fold enrichment is defined as the EASE score of the current module to the geometric mean of EASE scores in all modules (Hosack et al., 2003). The gene module values of all the *n* = 44 samples were represented as a gene module matrix ${\text{B}}_{M}\in {\mathbb{R}}^{m_{1}\times n}$. The *i*-th gene module value in *j*-th sample, (**B**_*M*_)_*i,j*_, was calculated by taking the sum of expressions of all the genes in the *i*-th module. Then **B**_*M*_ was rescaled row-wise by taking the *z*-scores across samples to compensate for the effect of variable module sizes.

#### 2.3.2. Cancer Pathways

Although the gene module representation is able to capture the variances across samples and reduce the redundancy of raw gene expressions, it has two disadvantages. The first is a lack of interpretability. Specifically, some annotations assigned by DAVID are not directly related to biological functions, and the annotations of different modules may substantially overlap. The second is that the key perturbed cancer pathways or functions may not always be the ones that vary most across samples. For example, genes in cancer-related KEGG pathways (hsa05200; Kanehisa and Goto, 2000) are not especially enriched in the top 3,000 genes with the largest expression variances. To make better use of prior knowledge on cancer-relevant pathways, we supplemented the generic DAVID pathway sets with a KEGG “cancer pathway” representation of samples ${\text{B}}_{P}\in {\mathbb{R}}^{m_{2}\times n}$, where the number of cancer pathways *m*_2_ = 24. The cancer-related pathways in the KEGG database are cleaner and easier to explain, more orthogonal to each other, and contain critical signaling pathways to cancer development. We extracted the 23 cancer-related pathways from the following 3 KEGG pathway sets: *Pathways in cancer* (hsa05200), *Breast cancer* (hsa05224), and *Glioma* (hsa05214). An additional cancer pathway *RET pathway* was added, since it was found to be recurrently gained in the prior research (Vareslija et al., 2018). See *y*-axis of **Figure 4D** for the complete list of 24 cancer pathways. We considered all the ~20,000 protein-coding genes other than top 3,000 genes. The following mapping of cancer pathways and transformation to *z*-scores were similar to that we did to map the gene modules.

Until this step, the raw gene expressions of *n* samples were transformed into the compressed gene module/pathway representation of samples $\text{B}={\left[\begin{array}{c} {\text{B}}_{M}^{⊺},{\text{B}}_{P}^{⊺} \end{array}\right]}^{⊺}\in {\mathbb{R}}^{m\times n}$, where *m* = *m*_1_ + *m*_2_. The gene module representation **B**_*M*_ serves for accurately deconvolving and unmixing the cell communities, while the pathway representation **B**_*P*_ serves as markers/probes and for interpretation purpose.

### 2.4. Deconvolution of Bulk Data

We applied a type of matrix factorization (MF) with constraints on the pathway-level expression signatures to deconvolve the communities/populations from primary and metastatic tumor samples (Figure 1, Step 2) (Koren et al., 2009). Note that common alternatives, such as principal components analysis (PCA) and non-negative matrix factorization (NMF) are not amenable to this case (Lee and Seung, 2000), since PCA does not provide a feasible solution to the constrained problem, and the NMF does not apply to our mixture data, which can be either positive or negative.

#### 2.4.1. Cell Communities

We define a cell community to be a set of clones/clonal subpopulations and other cell types that propagate as a group during the evolution of a tumor. A community may be just a single subpopulation/clone, but is a more general concept in the sense that it usually involves multiple related clones and their associated stroma. For example, a set of immunogenic clones and the immune cells infiltrating them might collectively form a community that has a collective expression signature mixing signatures of the clones and associated immune cells, even if the individual cell types are not distinguishable from bulk expression data alone. While much work in this space has classically aimed to separate individual clones, or perhaps individual cell types more broadly defined, we note that deconvolution may be unable in principle to resolve distinct cell types if they are always co-located in similar proportions. It is particularly true when data is sparse and cell types are fit only approximately, as in the present work, that a model with large complexity to deconvolve the fine-grained populations is prone to overfit. The community concept is intended in part to better describe the results we expect to achieve from the kind of data examined here and in part because identifying these communities is itself of interest in understanding how tumor cells coevolve with their stroma during progression and metastasis. Single-cell methods may provide an alternative, but are not amenable to preserved samples, such as are needed when retrospectively studying primary tumors and metastases that may have been biopsied years apart.

#### 2.4.2. Formulation of Deconvolution

With a matrix of bulk pathway values **B** ∈ ℝ^*m*×*n*^, the deconvolution problem is to find a component matrix $\text{C}={\left[\begin{array}{c} {\text{C}}_{M}^{⊺},{\text{C}}_{P}^{⊺} \end{array}\right]}^{⊺}\in {\mathbb{R}}^{m\times k}$ that represents the inferred fundamental communities of tumors, and the corresponding set of mixture fractions $\text{F}\in {\mathbb{R}}_{+}^{k\times n}$:

(1) $$\begin{array}{rc} \underset{\text{C},\text{F}}{\min}\; & ||\text{B}-\text{C}\text{F}|{|}_{\text{Fr}}^{2}, \end{array}$$

(2) $$\begin{array}{rcl} \text{s.t. } & {\text{F}}_{lj}\geq 0, & l=1,...,k,\;j=1,...,n, \end{array}$$

(3) $$\begin{array}{rc} \sum_{l=1}^{k}{\text{F}}_{lj}=1, & j=1,...,n, \end{array}$$

where ||**X**||_Fr_ is the Frobenius norm. The column-wise normalization in Equation (3) aims for recovering the biologically meaningful cell communities. In addition, they are equivalent to applying ℓ_1_ regularizers and therefore enforce sparsity to the fraction matrix **F** (Supplementary Material).

#### 2.4.3. Neural Network Deconvolution

**Figure 2:**
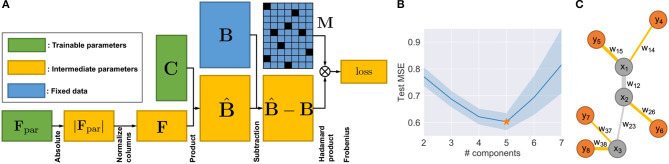
Method details. **(A)** Neural network architecture of NND. **(B)** Test errors of NND using 20-fold CV. Errors in unit of mean square error (MSE). **(C)** Illustration of a phylogeny with five extant nodes and three Steiner nodes.

Although it is possible to build new algorithms for solving MF by adapting previous work (Lee and Seung, 2000), the additional but necessary constraints of Equations (2) and (3) make the optimization much harder to solve. For the problem of Equations (1)–(3), one can prove that it does not generally guarantee convexity (Supplementary Material). A slightly modified version of the algorithm to solve NMF with constraints may guarantee neither good fitting nor convergence (Lei et al., 2019, 2020). Therefore, instead of revising existing MF algorithms, such as ALS-FunkSVD (Funk, 2006; Bell and Koren, 2007; Koren et al., 2009), we developed an algorithm which we call “neural network deconvolution” (NND) to solve the optimization problem using gradient descent. Specifically, the NND was implemented using backpropagation in the form of a neural network (Figure 2A) with the PyTorch package (https://pytorch.org/) (Rumelhart et al., 1986; Kingma and Ba, 2014), based on the revised constraints:

(4) $$\begin{array}{rc} \underset{\text{C},{\text{F}}_{\text{par}}}{\min}\; & ||\text{B}-\text{C}\text{F}|{|}_{\text{Fr}}^{2}, \end{array}$$

(5) $$\begin{array}{rc} \text{s.t. } & \text{F}=\text{cwn}\left(\left|{\text{F}}_{\text{par}}\right|\right), \end{array}$$

where |**X**| applies element-wise absolute value and cwn(**X**) is column-wise normalization, so that each column sums up to 1. The two operations of Equation (5) naturally rephrase and remove the two constraints in Equations (2) and (3), and meanwhile fit the framework of neural networks. An alternative to the absolute value operation |**X**| might be rectified linear unit ReLU(**X**) = max(**0**, **X**). However, this activation function is unstable and leads to inferior performance in our case, since **X**_*lj*_ will be fixed to zero once it becomes negative and will lose the chance to get updated in the following iterations. One may also want to replace the column-wise normalization cwn(**X**) with softmax operation softmax(**X**). However, the non-linearity introduced by softmax actually changes the original optimization problem (Equations 1–3) and the fitted **F** is therefore not sparse.

Based on the revised NND optimization problem (Equations 4 and 5), we built the neural network with the architecture shown in Figure 2A. An Adam optimizer other than vanilla gradient descent was used with default momentum parameters β_1_ = 0.9, β_2_ = 0.999 and learning rate of 1 × 10^−5^ (Kingma and Ba, 2014). The mini-batch technique is not required since the data size in our application is small enough not to require it (**B** ∈ ℝ^*m*×*n*^, *m* = 133, *n* = 44). The training is run until convergence, which is defined as when the relative decrease of training loss is smaller than ϵ = 1 × 10^−10^ every 20,000 iterations. This implementation has two main advantages: First, the method can be easily adapted to a wide range of optimization scenarios with various constraints, when existing methods do not or are hard to apply. Second, the NND has the flexibility of allowing for cross-validation, which is important for us in choosing the number of components *k* and preventing overfitting.

One might be suspicious whether the neural network fits precisely in practice, since it is based on a simple gradient descent optimization. To validate the fitting ability of NND, we plotted the PCA of original samples **B** and the fitted samples $\hat{\text{B}}$ (Supplementary Material). One can easily see that NND provides a good fit to the data.

#### 2.4.4. Cross-Validation of NND

In order to find the best tradeoff between model complexity and overfitting, we used cross-validation (CV) with the “masking” method to choose the optimal number of components/communities *k* = 5 that has the smallest test error (Figure 2B). In each fold of the CV, we used estimated $\hat{\text{B}}$ to only fit some randomly selected elements of **B**, and then the test error was calculated using the other elements of **B**. This was implemented by introducing two additional mask matrices ${\text{M}}_{\text{train}},{\text{M}}_{\text{test}}\in {\left\{0,1\right\}}^{m\times n}$, which are in the same shape of **B**, and ${\text{M}}_{\text{train}}+{\text{M}}_{\text{test}}=1^{m\times n}$. During the training time, with the same constraints in Equation (5), the optimization goal is:

(6) $$\begin{array}{r} \underset{\text{C},{\text{F}}_{\text{par}}}{\min}||{\text{M}}_{\text{train}}⊙\left(\text{B}-\text{C}\text{F}\right)|{|}_{\text{Fr}}^{2}, \end{array}$$

where **X** ⊙ **Y** is the Hadamard (element-wise) product. At the time of evaluation, given optimized $\hat{\text{C}}$, ${\hat{\text{F}}}_{\text{par}}$, and therefore optimized $\hat{\text{F}}=\text{cwn}\left(\left|{\hat{\text{F}}}_{\text{par}}\right|\right)$ for the optimization problem during training, the test error was calculated on the test set: $||{\text{M}}_{\text{test}}⊙\left(\text{B}-\hat{\text{C}}\hat{\text{F}}\right)|{|}_{\text{Fr}}^{2}$. We used 20-fold cross-validation on the NND, so in each fold 95% of positions of **M**_train_ and 5% of positions of **M**_test_ were 1s. Note that the actual number of cell populations is probably considerably larger than 5, and therefore each one of the five communities may contain multiple cell populations. Furthermore, it is likely that with sufficient numbers and precision of measurements, these communities could be more finely resolved into their constituent cell types. However *k* = 5 represents the largest hypothesis space of NND model that can be applied to the current dataset without severe overfitting.

### 2.5. Phylogeny of Inferred Cell Subcommunities and Pathway Inference of Steiner Nodes

We built “phylogenies” of cell subcommunities and estimated the pathway representation of unobserved (Steiner) nodes (Lu et al., 2003) inferred to be ancestral to them, with the goal of discovering critical communities that appear to be involved in the transition to metastasis and identifying the important changes of functions and expression pathways during this transition (Figure 1, Step 3). Note that we are using the term “phylogeny” loosely here, as these trees are intended to capture evolution of populations of cells not just by accumulation of mutations from a single ancestral clone but also via changes in community structure, for example, due to generating or suppressing an immune response or migrating to a metastatic site. Although an abuse of terminology, we use the term phylogeny here due to the methodological similarity to more proper phylogenetic methods in wide use for analyzing mutational data in cancers (Schwartz and Schäffer, 2017).

#### 2.5.1. Phylogeny of Communities

Given the pathway profiles of the extant communities at the time of collecting tumor samples **C** ∈ ℝ^*m*×*k*^, a phylogeny of the *k* extant cell communities was built using the neighbor-joining (NJ) algorithm (Nei and Saitou, 1987), which inferred a tree that contains *k* extant nodes/leaves, *k*−2 unobserved Steiner nodes, and edges connecting two Steiner nodes or a Steiner node and an extant node. We estimated an evolutionary distance for any pair of two communities *u*, *v* as the input of NJ using the Euclidean distance between their pathway vectors ||**C**_·*u*_ − **C**_·*v*_||_2_, similar to that in a prior work (Park et al., 2009).

#### 2.5.2. Inference of Pathways: Setting and Approach

Denote the phylogeny of cell subcommunities as G=(V,E), and $V=V_{S}\cup V_{C}$, where the indices of Steiner node $V_{S}=\left\{1,2,...,k-2\right\}$ ($|V_{S}|=k-2$), the indices of extant nodes $V_{C}=\left\{k-1,k,...,2k-2\right\}$ ($|V_{C}|=k$). For each edge (u,v)∈E, where 1 ≤ *u* < *v* ≤ 2*k*−2, the first node of edge *u* ≤ *k*−2 is always a Steiner node. The second node *v* can be either a Steiner node (*v* ≤ *k*−2) or extant node (*v* ≥ *k* − 1). Denote the set of weights $W=\left\{w_{uv}=1/d_{uv}\;|\;\left(u,v\right)\in E\right\}$ (inverse distance), where the edge length *d*_*uv*_ is the output of NJ. For each dimension *i* of the pathway vectors, we consider them independently and separately, so that each dimension of the Steiner nodes can be solved in the same way. Now let us consider the *i*-th dimension (and omit the subscript *i* for brevity) of extant nodes $V_{C}$: $\text{y}={\left[y_{k-1},y_{k},...,y_{2k-2}\right]}^{⊺}={\text{C}}_{i\cdot }^{⊺}\in {\mathbb{R}}^{k}$ and Steiner nodes $V_{S}$: $\text{x}={\left[x_{1},x_{2},...,x_{k-2}\right]}^{⊺}\in {\mathbb{R}}^{k-2}$. Figure 2C illustrates a phylogeny where *k* = 5. The inference of the *i*-th element in the pathway vector of the Steiner nodes can be formulated as minimizing the following elastic potential energy U(x,y;W):

(7) $$\begin{array}{l} \underset{\text{x}}{\min}\;U\left(\text{x},\text{y};W\right)=\underset{\begin{array}{c} \left(u,v\right)\in E\\ v\leq k-2 \end{array}}{\sum }\frac{1}{2}w_{uv}{\left(x_{u}-x_{v}\right)}^{2}\\ \;+\underset{\begin{array}{c} \left(u,v\right)\in E\\ v\geq k-1 \end{array}}{\sum }\frac{1}{2}w_{uv}{\left(x_{u}-y_{v}\right)}^{2}, \end{array}$$

which can be rephrased as a quadratic programming problem and solved easily, as we show below.

#### 2.5.3. Inference of Pathways: Derivation of Quadratic Programming, P(W), and q(W,y)

**Theorem 1**. *Equation (7) can be further rephrased as a quadratic programming problem*:

(8) $$\begin{array}{r} \underset{\text{x}}{\min}\;\frac{1}{2}{\text{x}}^{⊺}\text{P}\left(W\right)\text{x}+\text{q}{\left(W,\text{y}\right)}^{⊺}\text{x}, \end{array}$$

*where* P(W) *is a function that takes as input edge weights* W *and outputs a matrix* **P** ∈ ℝ^(*k*−2) × (*k*−2)^, q(W,y) *is a function that takes as input edge weights* W *and vector* **y** *and outputs a vector* **q** ∈ ℝ^*k*−2^.

Proof: Based on Equation (7), U(x,y;W)≥0. Each term inside the first summation (*v* ≤ *k* − 2) can be written as:

(9) $$\begin{array}{r} \frac{1}{2}w_{uv}{\left(x_{u}-x_{v}\right)}^{2}=\frac{1}{2}{\text{x}}^{⊺}\text{P}\left(w_{uv}\right)\text{x}, \end{array}$$

where

(10) $$P(w_{uv})=\begin{array}{c} \;\\ u\text{-th row }\\ \;\\ v\text{-th row }\\ \; \end{array}\overset{\begin{array}{c} \begin{array}{cc} u\text{-th col} & \;v\text{-th col} \end{array} \end{array}}{\left[\begin{array}{ccccc} 0 & 0 & 0 & 0 & 0\\ 0 & w_{uv} & 0 & -w_{uv} & 0\\ 0 & 0 & 0 & 0 & 0\\ 0 & -w_{uv} & 0 & w_{uv} & 0\\ 0 & 0 & 0 & 0 & 0 \end{array}\right]}.$$

Each term (*v* ≥ *k* − 1) inside the second summation can be rephrased as:

(11) $$\begin{array}{r} \frac{1}{2}w_{uv}{\left(x_{u}-y_{v}\right)}^{2}=\frac{1}{2}{\text{x}}^{⊺}\text{P}\left(w_{uv}\right)\text{x}+\text{q}{\left(w_{uv},y_{v}\right)}^{⊺}\text{x}+C\left(w_{uv},y_{v}\right), \end{array}$$

where

(12) $$\begin{array}{l} P(w_{uv})=\begin{array}{c} \;\\ \;u\text{-th row }\\ \;\\ \;\\ \; \end{array}\overset{\begin{array}{c} \;u\text{-th col} \end{array}\;}{\left[\begin{array}{ccccc} 0 & 0 & 0 & 0 & 0\\ 0 & w_{uv} & 0 & 0 & 0\\ 0 & 0 & 0 & 0 & 0\\ 0 & 0 & 0 & 0 & 0\\ 0 & 0 & 0 & 0 & 0 \end{array}\right]},\\ \;q(w_{uv},y_{v})=\begin{array}{c} \;\\ \;u\text{-th row }\\ \;\\ \;\\ \; \end{array}\overset{\begin{array}{c} \; \end{array}}{\left[\begin{array}{c} 0\\ -w_{uv}y_{v}\\ 0\\ 0\\ 0 \end{array}\right]}, \end{array}$$

and $C\left(w_{uv},y_{v}\right)=\frac{1}{2}w_{uv}y_{v}^{2}$ is independent of **x**. Therefore the optimization in Equation (7) can be calculated and written as below:

(13) $$\begin{array}{l} \underset{\text{x}}{\min}\underset{\begin{array}{c} \left(u,v\right)\in E\\ v\leq k-2 \end{array}}{\sum }\frac{1}{2}{\text{x}}^{⊺}\text{P}\left(w_{uv}\right)\text{x}\\ \;+\underset{\begin{array}{c} \left(u,v\right)\in E\\ v\geq k-1 \end{array}}{\sum }\left(\frac{1}{2}{\text{x}}^{⊺}\text{P}\left(w_{uv}\right)\text{x}+\text{q}{\left(w_{uv},y_{v}\right)}^{⊺}\text{x}\right), \end{array}$$

(14) $$\begin{array}{l} \Leftrightarrow \underset{\text{x}}{\min}\frac{1}{2}{\text{x}}^{⊺}\left(\underset{\begin{array}{c} \left(u,v\right)\in E\\ v\leq k-2 \end{array}}{\sum }\text{P}\left(w_{uv}\right)+\underset{\begin{array}{c} \left(u,v\right)\in E\\ v\geq k-1 \end{array}}{\sum }\text{P}\left(w_{uv}\right)\right)\text{x}\\ \;+\underset{\begin{array}{c} \left(u,v\right)\in E\\ v\geq k-1 \end{array}}{\sum }\text{q}{\left(w_{uv},y_{v}\right)}^{⊺}\text{x}, \end{array}$$

(15) $$\begin{array}{r} \Leftrightarrow \underset{\text{x}}{\min}\frac{1}{2}{\text{x}}^{⊺}\text{P}\left(W\right)\text{x}+\text{q}{\left(W,\text{y}\right)}^{⊺}\text{x}.\;□ \end{array}$$

**Remark 1**. *The optimal **x**^⋆^ of the Equation (7), or the solution to the quadratic programming problem Equation (8) can be solved by setting the gradient to be* **0**:

(16) $$\begin{array}{r} \text{P}\left(W\right){\text{x}}^{⋆}+\text{q}\left(W,\text{y}\right)=0. \end{array}$$

*Therefore*,

(17) $$\begin{array}{r} {\text{x}}^{⋆}=-\text{P}{\left(W\right)}^{-1}\text{q}\left(W,\text{y}\right). \end{array}$$

**Remark 2**. *Based on the proof, we can derive how to calculate the matrix* P(W) *and vector* q(W,y).

*Initialize the matrix and vector with zeros*:

(18) $$\begin{array}{r} \text{P}\leftarrow 0^{\left(k-2\right)\times \left(k-2\right)},\;\text{q}\leftarrow 0^{k-2}. \end{array}$$

*For each edge* (u,v)∈E with weight *w*_*uv*_, *there are two possibilities of nodes* *u* *and* *v*: *First, if both of them are Steiner nodes* (*u* ≤ *k*−2, *v* ≤ *k*−2), *we update* **P** *and keep* **q** *the same*:

$$\begin{array}{r} {\text{P}}_{uu}\leftarrow {\text{P}}_{uu}+w_{uv},{\text{P}}_{vv}\leftarrow {\text{P}}_{vv}+w_{uv}, \end{array}$$

(19) $$\begin{array}{r} {\text{P}}_{uv}\leftarrow {\text{P}}_{uv}-w_{uv},{\text{P}}_{vu}\leftarrow {\text{P}}_{vu}-w_{uv}. \end{array}$$

*Second, if u is Steiner node and v is an extant node* (*u* ≤ *k* − 2, *v* ≥ *k* − 1), *we update both* **P** *and* **q**:

(20) $$\begin{array}{r} {\text{P}}_{uu}\leftarrow {\text{P}}_{uu}+w_{uv},\;{\text{q}}_{u}\leftarrow {\text{q}}_{u}-y_{v}\cdot w_{uv}. \end{array}$$

*We apply the same procedure to all dimension of pathways* *i* = 1, 2, ..., *m* *to get the full pathway values for each Steiner node*.

## 3. Results

### 3.1. NND Deconvolves the Bulk RNA Accurately

Before we applied our deconvolution algorithm NND to the breast cancer brain metastatic samples, we first validated our algorithm on a semi-simulated dataset where the ground truth expressions and fractions of each cell clone in the mixture samples are known.

#### 3.1.1. Semi-simulated GSE11103 Dataset

The semi-simulated dataset is based on the real data of pure clones from the GSE11103 dataset (Abbas et al., 2009; Barrett et al., 2013). Expression profiles of four different cells were measured using microarrays: Raji (B cell), IM-9 (B cell), THP-1 (monocyte), Jurkat (T cell). Each experiment was repeated three times. We took the average of the three replicates to get the expression data of the four pure cell clones. The top 300 genes that varied most across cell types were selected as the ground truth real data of pure cell clones: $\text{C}\in {\mathbb{R}}_{+}^{300\times 4}$. We then created 100 mixture samples of the four pure clones *in silico* $\text{B}\in {\mathbb{R}}_{+}^{300\times 100}$ by randomly generating the fraction matrix $\text{F}\in {\mathbb{R}}_{+}^{4\times 100}$. The fraction matrix was generated in the following way:

(21) $$\begin{array}{r} {\text{F}}_{lj}\leftarrow U\left(0,1\right),\;l=1,...,4,\;j=1,...,100, \end{array}$$

(22) $$F_{lj}\leftarrow \frac{F_{lj}}{\sum_{l^{\prime }=1}^{4}F_{l^{\prime }j}},\;j=1,\;...,\;100,$$

where *U*(0, 1) is a uniform distribution in the interval [0, 1]. The semi-simulated bulk expression matrix **B** was then generated from **C**, **F**, with a log-normal noise:

(23) $$\begin{array}{ll} {\left(\text{B}\right)}_{ij}={\left(\text{C}\text{F}\right)}_{ij}+2^{N\left(0,{\left(s\sigma \right)}^{2}\right)}, & \;i=1,...,300,\;j=1,...,100, \end{array}$$

where $N\left(0,{\left(s\sigma \right)}^{2}\right)$ is a Gaussian distribution; *s* controls the noise level, which we set to 0, 0.4, 0.9, and 1.3 for test; σ is the standard deviation of log_2_-transformed original GSE11103 data.

#### 3.1.2. Performance Evaluation

Given the bulk matrix **B**, we applied NND and other two algorithms to infer the estimated $\hat{\text{C}}$, $\hat{\text{F}}$ and $\hat{\text{B}}=\hat{\text{C}}\hat{\text{F}}$, and compared the accuracy between estimated and actual values using the following metrics. For **C**, we used *L*_1_ loss (Zhu et al., 2018):

(24) $$\begin{array}{r} L_{1}\;\text{loss}\left(\text{C}\right)=\frac{||\hat{\text{C}}-\text{C}|{|}_{1}}{||\text{C}|{|}_{1}}. \end{array}$$

For **F** and **B**, we used root mean square error (RMSE):

(25) $$\begin{array}{r} \text{RMSE}\left(\text{F}\right)=\sqrt{||\hat{\text{F}}-\text{F}|{|}_{\text{Fr}}^{2}}, \end{array}$$

(26) $$\begin{array}{r} \text{RMSE}\left(\text{B}\right)=\sqrt{\frac{||\hat{\text{B}}-\text{B}|{|}_{\text{Fr}}^{2}}{||\text{B}|{|}_{\text{Fr}}^{2}}} \end{array}$$

Different levels of noise *s* were added to test the robustness of models and the performance of different models under different conditions. We repeated all the experiments for 10 times to get the boxplot.

#### 3.1.3. Competing Algorithms

There are two competing algorithms for the deconvolution problem. Geometric unmixing is an algorithm that borrows the intuition from computational geometry (Schwartz and Shackney, 2010), which first identifies the corners of a simplex containing all the mixture sample points, and then infers the fraction matrix. However, the algorithm does not directly optimize the problem (Equations 1–3). Another intuitive algorithm is based on the popular multiplicative update (MU) rule that solves general NMF problem (Lee and Seung, 2000): an additional update step of $F_{lj}\;\leftarrow \frac{F_{lj}}{\sum_{l^{\prime }=1}^{k}F_{l^{\prime }j}},j=1,\;...,\;n$ can be added to the loop. Although the original MU rule guarantees the non-increasing of the objective function, this additional update step can lead to an increasing objective and we need to stop the iteration once this happened. Since the two competing algorithms work on non-negative space, we adapted the NND by adding an element-wise absolute value operator after the **C** in the network (Figure 2A).

#### 3.1.4. Superiority of NND

**Figure 3:**
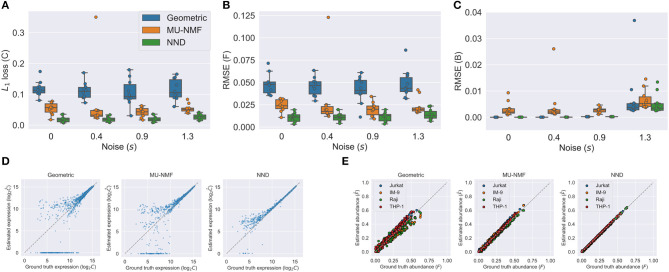
Comparison of NND and other algorithms on the semi-simulated GSE11103 dataset shows the better accuracy and robustness of NND algorithm. **(A–C)** Accuracies in estimated **C**, **F**, and **B** with three different algorithms. We tested four noise levels and repeated the experiments for ten replicates. **(D)** Estimated $\hat{\text{C}}$ and ground truth **C** using three different algorithms with noise level *s* of 1.3. **(E)** Estimated $\hat{\text{F}}$ and ground truth **F** using three algorithms with noise level *s* of 1.3.

We show the results in Figure 3. Figures 3A–C show the accuracies of both **C**, **F**, and **B** using the three algorithms under various noise levels. One can easily see that NND achieves lower *L*_1_ loss of **C**, RMSE of **F**, and RMSE of **B**. What is more, it is also much more robust than the geometric and MU-NMF algorithms, as there are fewer outliers that have huge errors. MU-NMF has a reasonable estimation accuracy of **C** and **F**. However, its overall fitting ability is limited due to its non-convergence-guaranteed MU optimization algorithm. We can also visualize the estimation accuracy by plotting the estimated values and ground truth values at a specific noise level, as is shown in Figures 3D,E. One can see the superiority of NND qualitatively over the other two algorithms in estimating expression profiles and fractions of individual pure clones.

### 3.2. Gene Modules/Pathways Provide an Effective Representation

Gene expressions of samples were mapped into the gene module and pathway space in order to reduce the noise of raw transcriptome data and reduce redundancy (section 2.3). We verified that the gene module/pathway representation is effective in the sense that it captures distinguishing features of primary/metastatic sites and individual samples well and is able to identify recurrently gained or lost pathways.

#### 3.2.1. Feature Space of the Gene Module and Pathway Representation

**Figure 4:**
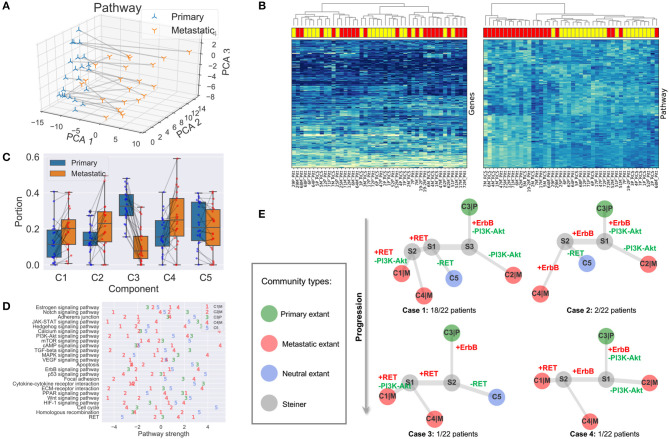
Results and analysis. **(A)** First three gene module/pathway representation PCA dimensions of matched primary and metastatic samples. Matched samples are connected. **(B)** Hierarchical clustering of tumor samples based on raw gene expressions (left panel) and compressed gene module/pathway representation (right panel). Metastatic samples are shown in red rectangles and primary ones in yellow. **(C)** Portions and changes of the five communities in primary and metastatic sites. Each gray line connects the portions of a community in the primary site (blue node) and metastatic site (red node) from the same patient. **(D)** Pathway strengths across cell communities. **(E)** Phylogeny of cell subcommunities.

As one can see in Figure 4A, the first principal component analysis (PCA) dimension of the gene module and pathway representation accounts for the difference between primary and metastatic samples, while the second and third PCA dimensions mainly capture variability between patients. This observation suggests the feasibility of using the gene module/pathway representation to distinguish recurrent features of metastatic progression across patients despite heterogeneity between patients. To make a direct comparison of the noise and redundancy between the gene module/pathway and raw gene expression representations, we applied hierarchical clustering to the 44 samples using Ward's minimum variance method (Ward, 1963). Two hierarchical trees were built based on the two different representations (Figure 4B). The gene module/pathway features more effectively separate the primary and metastatic samples into distinct clusters (Figure 4B, right panel) than do the raw gene expression values (Figure 4B, left panel). This is consistent with the PCA results that the largest mode of variance in the pathway representation distinguishes primary from metastatic samples. We do notice that in a few cases, matched primary and metastatic samples from the same patient are neighbors with pathway-based clustering. For example, 29P_Pitt:29M_Pitt and 51P_Pitt:51M_Pitt are grouped in the same clades using the pathway representation, showing that in a minority of cases, features of individual patients dominate over primary vs. metastatic features. Following previous work (Park et al., 2009), we quantified the ability of the hierarchical tree to group the samples of the same labels using four metrics. (1) MSD: Mean square distance of edges that connect nodes of the same label (primary vs. metastatic). (2) *z*_MSD_: The labels of all nodes were shuffled and the MSD is recalculated for 1,000 times to get the mean μ_MSD_ and standard deviation σ_MSD_, which were used to get the *z*-score of the current assignment *z*_MSD_ = (MSD−μ_MSD_)/σ_MSD_. (3) rMSD: The ratio of MSD of edges that connect same label nodes and MSD of edges that connect distinct label nodes. (4) *z*_rMSD_: as with MSD, a *z*-score of rMSD was calculated by shuffling labels for 1,000 times. Intuitively, the smaller values the MSD, *z*_MSD_, rMSD, and *z*_rMSD_ are, the better is the feature representation at grouping same label samples together. The shortest paths and distances between all pairs of nodes were calculated using the Floyd-Warshall algorithm (Floyd, 1962; Warshall, 1962). All the edge lengths were considered as 1.0 to account for the different scales of pathway and gene representations. The pathway representation has significantly lower values for all four metrics (Table 1), indicating its strong grouping ability.

**Table 1:** Quantitative performance of hierarchical clustering.

**Feature representation**	**MSD**	**rMSD**	***z*_MSD_**	***z*_rMSD_**
Gene expression	99.62	0.93	−2.60	−2.57
Gene module/pathway	86.23	0.66	−13.37	−11.42

#### 3.2.2. Recurrently Perturbed Cancer Pathways

We next identified differentially expressed pathways in the primary and metastatic tumors using bulk data ${\text{B}}_{P}\in {\mathbb{R}}^{24\times 44}$, prior to deconvolving cellular subcommunities. We conducted the Student's *t*-test followed by FDR correction on each of the 24 pathways. Eleven pathways are significantly different between the two sites (FDR < 0.05; Table 2). The signaling pathways related to neurotransmitter and calcium homeostasis, including *cAMP* and *Calcium* (Hofer and Lefkimmiatis, 2007), are enriched in metastatic samples, which we can suggest may reflect stromal contamination by neural cells in the brain metastatic samples. We also observed recurrent gains in *ErbB* pathway, as indicated by the primary studies (Priedigkeit et al., 2017; Vareslija et al., 2018). Three pathways related to immune activity are under-expressed in metastatic samples, including *Cytokine-cytokine receptor interaction* (Lee and Margolin, 2011), *JAK-STAT* (Lee and Margolin, 2011), and *Notch* (Aster et al., 2017), consistent with the previous inference of reduced immune cell expression in metastases in general and brain metastasis most prominently (Zhu et al., 2019). We can suggest that this result similarly may reflect expression changes in infiltrating immune cells, due to the immunologically privileged environment of the brain, rather than expression changes in tumor cell populations. Five other signaling pathways, including *Apoptosis* (Wong, 2011), *Wnt* (Zhan et al., 2016), *Hedgehog* (Gupta et al., 2010), *PI3K-Akt* (Brastianos et al., 2015), and *TGF-beta* (Massagué, 2008), show reduction in metastatic samples and in each case, their loss or dysregulation has been reported to promote the tumor growth and brain metastasis. Note that the primary references for these data define pathways using the co-expression pattern of genes (Priedigkeit et al., 2017; Vareslija et al., 2018), while our work uses external knowledge bases. Previous research also used somatic mutations or copy number variation to analyze perturbed genes (Brastianos et al., 2015; Priedigkeit et al., 2017), while we focus exclusively on the transcriptome. Despite large differences in data types and pathway definitions, our observations are consistent with the prior analysis, especially with respect to variation in the *HER2/ErbB2* and *PI3K-Akt* pathways.

**Table 2:** Differentially expressed cancer pathways between primary and metastatic samples (FDR < 0.05).

**Gain/Loss after metastasis**	**Differentially expressed pathways**	**FDR**
Relative gain	cAMP signaling pathway	6.88e-03
Relative gain	ErbB signaling pathway	2.09e-02
Relative gain	Calcium signaling pathway	4.39e-02
Relative loss	Cytokine-cytokine receptor interaction	4.37e-06
Relative loss	Apoptosis	8.53e-04
Relative loss	JAK-STAT signaling pathway	8.53e-04
Relative loss	Wnt signaling pathway	3.97e-03
Relative loss	Hedgehog signaling pathway	4.50e-03
Relative loss	PI3K-Akt signaling pathway	1.35e-02
Relative loss	TGF-beta signaling pathway	4.56e-02
Relative loss	Notch signaling pathway	4.56e-02

### 3.3. Landscape of Deconvolved Cell Communities in Tumors

We unmixed the bulk data **B** into five components using NND (section 2.4). The deconvolution enables us to produce at least a coarse-grained landscape of major cell communities **C** and their distributions in primary and metastatic tumors **F**. The number of components (*k* = 5) was chosen through 20-fold cross-validation (section 2.4; Figure 2B). Although the true heterogeneity of the samples may be much larger, we fit *k* to provide a balance between excessively coarse-grained communities if *k* is too small vs. excessively high variance and thus unstable deconvolution if *k* is too large.

#### 3.3.1. Community Distributions Across Samples **F**

The portions of the five components in all the 44 samples are represented as the mixture fraction matrix **F** ∈ ℝ^5 × 44^ (Figure 4C). A primary or metastatic community is one inferred to change proportions substantially (magnitude > 0.05) in the tumor samples after metastasis, or perhaps to be entirely novel to or extinct in the metastatic sample (denoted by a |*P* or |*M* suffix). Otherwise, the component is classified as a neutral community. Three components (*C*1|*M*, *C*2|*M*, *C*4|*M*) are classified as metastatic communities; one (*C*3|*P*) as primary; and one (*C*5) as neutral (Figure 4C). Some components may be missing in both samples of some patients, e.g., *C*1|*M*, *C*2|*M*, *C*5|*M* are absent in two, one, and one patient. We note that these five communities represent rough consensus clusters of cell populations inferred to occur frequently, but not universally, among the samples. Based on this rule, we can define four basic cases of patients in total. Twelve subcases can be found using a more detailed classification method based on the existence of communities in both primary and metastatic samples (Supplementary Material).

#### 3.3.2. Pathway Values of Communities **C**

We are especially interested in the pathway part **C**_*P*_ of the cell community inferences, since it serves as the marker and provides results easier to interpret. The pathway values of five subcommunities using **C**_*P*_ provides a much more fine-grained description of samples (Figure 4D), compared with that in section 3.2, which is only able to distinguish the differentially expressed pathways in bulk samples. As noted in section 2.4, it is likely that true cellular heterogeneity is greater than the methods are able to discriminate and that communities inferred by our model may each conflate one or more distinct cell types and clones. We observe that the metastatic community *C*4|*M* most prominently contributes to the enrichment for functions related to neurotransmitter and ion transport, since its strongest pathways (*cAMP, Calcium*) are greatly enriched relative to those of the other four communities. We might interpret this community as reflecting at least in part stromal contamination from neural cells specific to the metastatic site. *C*4|*M* also contributes most to the gains of *ErbB* in brain samples. The metastatic subcommunity *C*1|*M* is probably most closely related to the loss of immune response in metastatic samples as it has the lowest pathway values of *Notch, JAK-STAT*, and *Cytokine-cytokine receptor interaction*. This component might thus in part reflect the effect of relatively greater immune infiltration in the primary vs. the metastatic site. *C*1|*M* also has the lowest pathway values of *Apoptosis, Wnt*, and *Hedgehog*. The metastatic community *C*2|*M* is most responsible for the loss of *PI3K-Akt* and *TGF-beta* pathways. We also note that although *RET* does not show up in the list of Table 2, it seems to be quite over-expressed in the metastatic communities *C*1|*M* and *C*4|*M* but not in the metastatic community *C*2|*M*.

### 3.4. Phylogenies of BrM Communities Reveal Common Order of Perturbed Pathways

We built phylogenies of cell communities and calculated the pathway representations of their Steiner nodes (section 2.5). The phylogenies' topologies provide a way to infer a likely evolutionary history of cancer cell communities and thus their constitutive cell types. At the same time, the perturbed pathways along their edges suggest the order of genomic alterations or changes in community composition.

#### 3.4.1. Topologically Similar BrM Phylogenies

All five cell components do not appear in each BrM patient. We analyze the distribution of communities in each patient based on whether the community is inferred to be present in the patient (Supplementary Material). There are four different cases in general (Figure 4E). Case 1: all five communities are found in the patient (majority; 18/22 patients). Case 2: only *C*1|*M* missing (minority; 2/22). Case 3: only *C*2|*M* missing (minority; 1/22). Case 4: only *C*5 missing (minority; 1/22). Although not all communities exist in Cases 2–4, the topologies are similar to that of Case 1 and can be seen as special cases of Case 1, representing some inferred common mechanisms of progression across all the BrM patients.

#### 3.4.2. Common Order of Altered Cancer Pathways

After inferring the pathway values for Steiner nodes, the most perturbed pathways can also be found by subtracting the pathway vectors of nodes that share an edge. We focus on the top five gained or lost pathways along the evolutionary trajectories and the changes of magnitude larger than 1.0 (Supplementary Material). We further examine those perturbed cancer pathways that were specifically proposed in the study that generated the data examined here, as well as others that are clinically actionable (Brastianos et al., 2015; Priedigkeit et al., 2017; Vareslija et al., 2018), i.e., *ErbB, PI3K-Akt*, and *RET* (Figure 4E). As one may see from Case 1, the primary community *C*3|*P* first evolves to community *S*3 by gaining expressions in *ErbB* and losing functions in *PI3K-Akt*. Then, if it continues to lose *PI3K-Akt* activity, it will evolve into the metastatic community *C*2|*M*. If it gains in *RET* activity, it will instead evolve into metastatic communities *C*1|*M* and *C*4|*M*. The perturbed pathways along the trajectories of Cases 2–4 are similar to those of Case 1, with minor differences. We therefore draw to the conclusion that the evolution of BrMs follows a specific and common order of pathway perturbations. Specifically, the gain of *ErbB* reproducibly happens before the loss of *PI3K-Akt* and the gain of *RET*. Different subsequently perturbed pathways lead to different metastatic tumor cell communities. These inferences are consistent with the hypothesis that at least some major changes in expression programs between primary and metastatic communities occur by selecting for heterogeneity present early in tumor development rather than solely deriving from novel functional changes immediately prior to or after metastasis.

## 4. Discussion

Cancer metastasis is usually a precursor to mortality with no successful treatment options. Better understanding mechanisms of metastasis provides a potential pathway to identify new diagnostics or therapeutic targets that might catch metastasis before it ensues, treat it prophylactically, or provide more effective treatment options once it occurs. The present work developed a computational approach intended to better reconstruct mechanisms of functional adaption from multisite RNA-Seq data to help us understand at the level of cancer pathways the mechanisms by which progression frequently proceeds across a patient cohort. Our method compresses expression data into a gene module/pathway representation using external knowledge bases, deconvolves the bulk data into putative cell communities where each community contains a set of associated cell types or subclones, and builds evolutionary trees of inferred communities with the goal of reconstructing how these communities evolve, adapt, and reconfigure their compositions across metastatic progression. Results on semi-simulated data show the method to yield improved accuracy in mixture deconvolution relative to prior deconvolution algorithms. We applied the pipeline to matched transcriptome data from 22 BrM patients and found that although there are slight differences of tumor communities across the cohort, most patients share a similar mechanism of tumor evolution at the pathway level. Specifically, the methods infer a fairly conserved mechanism of early gain of *ErbB* prior to metastasis, followed post-metastasis gain of *RET* or loss of *PI3K-Akt* resulting in intertumor heterogeneity between samples. Our methods provide a novel way of viewing the development of BrM with implications for basic research into metastatic processes and potential translational applications in finding markers or drug targets of metastasis-producing clones prior to the metastatic transition.

The results suggest several possible avenues for future development. In part, they suggest a need for better separating phylogenetically-related mixture components (i.e., distinct tumor cell clones) from unrelated infiltrating cell types (e.g., healthy stroma from the primary or metastatic site or infiltrating immune cells). The methods are likely finding only a small fraction of the true clonal heterogeneity of the tumors and stroma, and might benefit from algorithms capable of better resolution or from integration of multi-omics data (e.g., RNA-Seq, DNA-Seq, methylation) that might have complementary value in finer discrimination of cell types. The present methods are also using only a limited form of temporal constraint in considering a two-stage progression process and without use of quantitative time measurements. Models might be extended in future work to consider true time-series data, such as is becoming available through “liquid biopsy” technologies. In addition, we know of no data with known ground truth that models the kind of progression process studied here nor of other tools designed for modeling similar progression processes from expression data, leaving us reliant on validating based on consistency with prior research on brain metastasis (Brastianos et al., 2015; Priedigkeit et al., 2017; Vareslija et al., 2018). Future work might compare to prior approaches for reconstruction of clonal evolution from expression data more generically (Desper et al., 2004; Riester et al., 2010; Schwartz and Shackney, 2010) and seek replication on additional real or simulated expression data or artificial mixtures of different cell types (Qiu et al., 2011) designed to mimic metastasis-like progression. The general approach might also have broader application than studying metastasis, for example in reconstructing mechanisms of other progression processes, such as pre-cancerous to cancerous, as well as to other tumor types or independent data sets. Finally, much remains to be done to exploit the translational potential of the method in better identifying diagnostic signatures and therapeutic targets, and what type of effective and safe clinical strategies can be taken to prevent metastasis at an early stage.

## Data Availability Statement

The breast cancer brain metastases dataset analyzed for this study can be found on Github: https://github.com/lizhu06/TILsComparison_PBTvsMET. The simulated dataset generated, and data for analysis for this study can be found on Github: https://github.com/CMUSchwartzLab/NND.

## Author Contributions

RS, JM, and AL contributed to the conceptualization. RS, JM, AL, YT, and HL contributed to the methodology. YT contributed to the software. RS, JM, AL, and YT contributed to the formal analysis, writing-review and editing, and funding acquisition. AL contributed to the resources. YT and HL contributed to the writing of the original draft. RS supervision. All authors contributed to the article and approved the submitted version.

## Conflict of Interest

The authors declare that the research was conducted in the absence of any commercial or financial relationships that could be construed as a potential conflict of interest.
