# Supplementary material for: Neural Network Deconvolution Method for Resolving Pathway-Level Progression of Tumor Clonal Expression Programs With Application to Breast Cancer Brain Metastases
Source: Front Physiol. 2020 Sep 4;11:1055. doi: 10.3389/fphys.2020.01055 (PMC7499245; doi:10.3389/fphys.2020.01055)
Supplement: Supplementary file 1 [file Data_Sheet_1.PDF]

Supplementary Material

1 NON-CONVEXITY OF DECONVOLUTION PROBLEM

THEOREM 1. *The deconvolution problem equation below is not convex:*

$$\min_{\mathbf{C},\mathbf{F}} \quad f(\mathbf{C},\mathbf{F}) = \|\mathbf{B} - \mathbf{CF}\|_{\mathrm{Fr}}^2, \tag{S1}$$

$$\textit{s.t.} \quad \mathbf{F}_{lj} \geq 0, \qquad l = 1, ..., k,\ j = 1, ..., n, \tag{S2}$$

$$\sum\nolimits_{l=1}^{k} \mathbf{F}_{lj} = 1, \qquad j = 1, ..., n. \tag{S3}$$

PROOF. If the problem is convex, we should have: $\forall \lambda \in (0, 1)$, and $\forall \mathbf{C}_x, \mathbf{C}_y, \mathbf{F}_x, \mathbf{F}_y$ in the feasible domain, the following inequality always holds:

$$\lambda f(\mathbf{C}_x, \mathbf{F}_x) + (1-\lambda) f(\mathbf{C}_y, \mathbf{F}_y) \geq f(\lambda \mathbf{C}_x + (1-\lambda)\mathbf{C}_y, \lambda \mathbf{F}_x + (1-\lambda)\mathbf{F}_y). \tag{S4}$$

However, for the following setting:

$$\mathbf{B} = \begin{bmatrix} -1.38 & 0.92 \\ 1.03 & -0.15 \end{bmatrix}, \tag{S5}$$

$$\mathbf{C}_x = \begin{bmatrix} -1.74 & 2.21 \\ 1.00 & -3.97 \end{bmatrix}, \qquad \mathbf{C}_y = \begin{bmatrix} 1.03 & -0.46 \\ -3.13 & 0.16 \end{bmatrix}, \tag{S6}$$

$$\mathbf{F}_x = \begin{bmatrix} 0.83 & 0.32 \\ 0.17 & 0.68 \end{bmatrix}, \qquad \mathbf{F}_y = \begin{bmatrix} 0.09 & 0.34 \\ 0.91 & 0.66 \end{bmatrix}, \tag{S7}$$

and $\lambda = 0.5$, we have

$$\lambda f(\mathbf{C}_x, \mathbf{F}_x) + (1-\lambda) f(\mathbf{C}_y, \mathbf{F}_y) = 4.86 < 11.74 = f(\lambda \mathbf{C}_x + (1-\lambda)\mathbf{C}_y, \lambda \mathbf{F}_x + (1-\lambda)\mathbf{F}_y). \tag{S8}$$

This is contradictory to equation (S4). □

2 SUPPLEMENTARY TABLES AND FIGURES

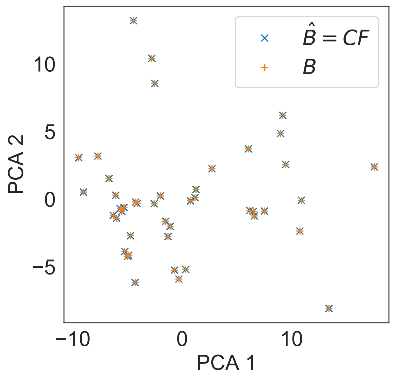

Figure S1. PCA of pathway representation B and NND fitted $\hat{\mathrm{B}}$. Each dot represents the pathway values of a sample $\mathrm{B}_{\cdot j}$ or fitted $\hat{\mathrm{B}}_{\cdot j}$. The first two PCA dimensions of original data and fitted data are almost in the same positions, which indicates that NND is able to fit precisely in our application. The number of components is set to be $k = 5$ here.

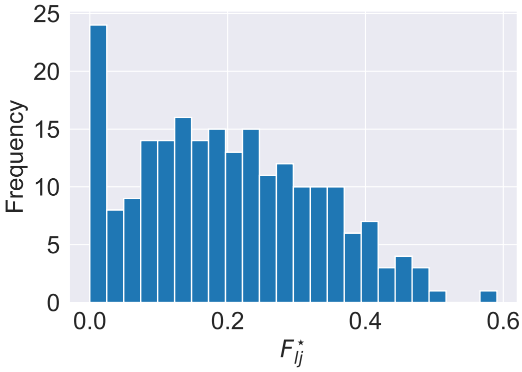

Figure S2. Sparsity of NND Results. We show the distribution of elements in NND deconvoluted fraction matrix $\mathbf{F}^{\star}$. Since each column of $\mathbf{F}$ is forced to sum up to be one, a Laplacian prior is applied to the elements of matrix $\mathbf{F}$. This leads to the sparsity of $\mathbf{F}^{\star}$: 24 out of its 220 elements ($k \times n = 5 \times 44$) are zeros (threshold set to 2.5×10^{-2}).

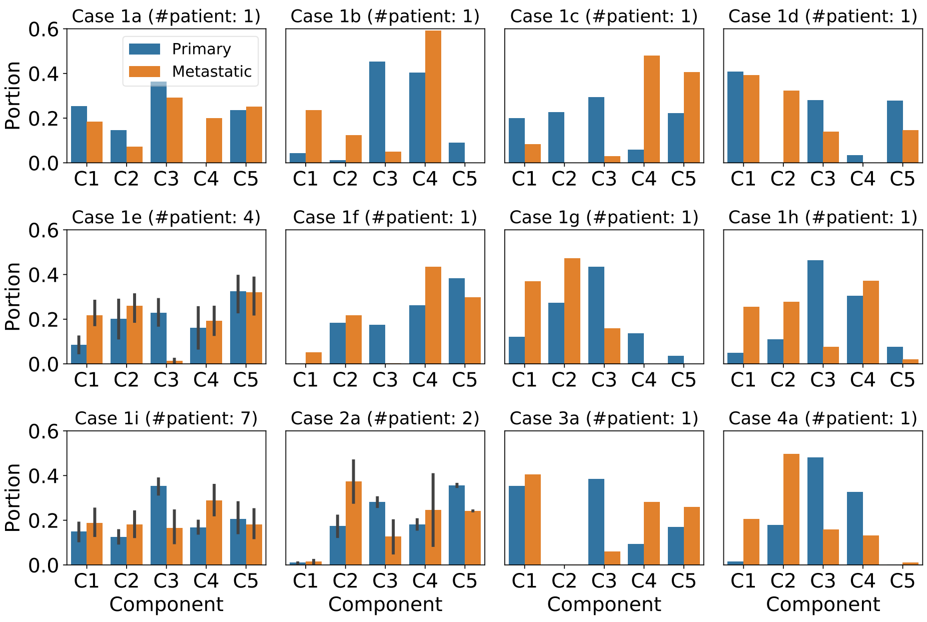

Figure S3. Classification of BrM patients based on the consisted cell subcommunities in matched samples. The figure displays, for each patient, the proportion of each community in the primary and the metastatic sample. There are 12 subcases of the 4 cases. Specifically, there are 9 specific cases (Case 1a-i) in Case 1. Most patients (7) have all five cell communities in both primary and metastatic samples (Case 1i). A few patients (4) have all communities in metastasis samples and all clones but community $C3|P$ in primary samples. The element $\mathbf{F}_{lj}$ is taken as 0 when it is smaller than a threshold 2.5×10^{-2}, and therefore the l-th community is missing in the j-th sample.

Table S1. Perturbed pathways during the evolution of cell communities in primary and metastatic tumors (Case 1). The top five perturbed pathways whose gain or loss greater than 1.0 along each edge of phylogeny are shown. Clinically actionable perturbed cancer pathways during metastasis are shown in boldface, i.e., *ErbB*, *RET*, and *PI3K-Akt*.

Trajectory	Gain	Perturbed Pathways	Loss	Perturbed Pathways
$C3\|P \rightarrow S3$	$+2.83$	Homologous recombination	-3.76	Hedgehog signaling pathway
	$+2.41$	Cell cycle	-3.45	Cytokine-cytokine receptor interaction
	$+1.86$	**ErbB signaling pathway**	-3.39	**PI3K-Akt signaling pathway**
	$+1.10$	cAMP signaling pathway	-3.15	TGF-beta signaling pathway
			-3.14	JAK-STAT signaling pathway
$S3 \rightarrow S1$	< 1.0	$\emptyset$	< 1.0	$\emptyset$
$S1 \rightarrow S2$	$+1.36$	cAMP signaling pathway	-1.28	JAK-STAT signaling pathway
	$+1.18$	**RET**	-1.22	Apoptosis
			-1.21	Cytokine-cytokine receptor interaction
			-1.12	Wnt signaling pathway
			-1.04	Notch signaling pathway
$S2 \rightarrow C1\|M$	$+1.90$	**RET**	-3.25	Wnt signaling pathway
	$+1.59$	PPAR signaling pathway	-3.11	JAK-STAT signaling pathway
			-2.77	Notch signaling pathway
			-2.48	Hedgehog signaling pathway
			-2.18	**PI3K-Akt signaling pathway**
$S2 \rightarrow C4\|M$	$+4.48$	Calcium signaling pathway	-3.06	p53 signaling pathway
	$+4.17$	cAMP signaling pathway	-2.74	Cell cycle
	$+3.83$	MAPK signaling pathway	-2.21	Homologous recombination
	$+3.35$	ECM-receptor interaction	-1.40	Apoptosis
	$+3.20$	Focal adhesion	-1.33	Cytokine-cytokine receptor interaction
$S1 \rightarrow C5$	$+3.91$	Cell cycle	-3.00	**RET**
	$+3.17$	p53 signaling pathway	-1.58	MAPK signaling pathway
	$+2.85$	Adherens junction	-1.41	cAMP signaling pathway
	$+2.76$	Cytokine-cytokine receptor interaction		
	$+2.68$	Wnt signaling pathway		
$S3 \rightarrow C2\|M$	$+1.39$	Homologous recombination	-3.65	TGF-beta signaling pathway
			-3.61	**PI3K-Akt signaling pathway**
			-3.34	ECM-receptor interaction
			-3.20	Focal adhesion
			-2.60	PPAR signaling pathway

Table S2. Perturbed pathways during the evolution of cell communities in primary and metastatic tumors (Case 2). The top five perturbed pathways whose gain or loss greater than 1.0 along each edge of phylogeny are shown.

Trajectory	Gain	Perturbed Pathways	Loss	Perturbed Pathways
$C3\|P \rightarrow S1$	$+2.83$	Homologous recombination	-3.22	Hedgehog signaling pathway
	$+2.47$	Cell cycle	-3.10	TGF-beta signaling pathway
	$+1.81$	**ErbB signaling pathway**	-3.08	Cytokine-cytokine receptor interaction
	$+1.02$	cAMP signaling pathway	-2.93	**PI3K-Akt signaling pathway**
			-2.64	PPAR signaling pathway
$S1 \rightarrow S2$	$+1.08$	ECM-receptor interaction		
	$+1.08$	**ErbB signaling pathway**		
$S2 \rightarrow C4\|M$	$+5.51$	cAMP signaling pathway	-3.97	Cell cycle
	$+5.12$	Calcium signaling pathway	-3.83	p53 signaling pathway
	$+4.45$	MAPK signaling pathway	-3.20	Apoptosis
	$+3.37$	ECM-receptor interaction	-3.15	Cytokine-cytokine receptor interaction
	$+3.08$	**ErbB signaling pathway**	-3.00	Homologous recombination
$S2 \rightarrow C5$	$+3.68$	Cell cycle	-2.25	**RET**
	$+3.18$	p53 signaling pathway	-1.81	MAPK signaling pathway
	$+2.50$	Homologous recombination	-1.43	cAMP signaling pathway
	$+2.16$	Adherens junction	-1.24	Hedgehog signaling pathway
	$+2.15$	Cytokine-cytokine receptor interaction	-1.13	Calcium signaling pathway
$S1 \rightarrow C2\|M$	$+1.39$	Homologous recombination	-4.06	**PI3K-Akt signaling pathway**
			-3.70	TGF-beta signaling pathway
			-3.55	Focal adhesion
			-3.52	ECM-receptor interaction
			-2.87	Adherens junction

Table S3. Perturbed pathways during the evolution of cell communities in primary and metastatic tumors (Case 3). The top five perturbed pathways whose gain or loss greater than 1.0 along each edge of phylogeny are shown.

Trajectory	Gain	Perturbed Pathways	Loss	Perturbed Pathways
$C3\|P \rightarrow S2$	$+3.10$	Cell cycle	-3.51	Hedgehog signaling pathway
	$+3.10$	**ErbB signaling pathway**	-2.41	Notch signaling pathway
	$+2.93$	Homologous recombination	-2.39	Cytokine-cytokine receptor interaction
	$+1.70$	cAMP signaling pathway	-2.34	JAK-STAT signaling pathway
	$+1.66$	HIF-1 signaling pathway	-2.07	Apoptosis
$S2 \rightarrow S1$	$+1.62$	cAMP signaling pathway	-2.02	Cytokine-cytokine receptor interaction
	$+1.54$	**RET**	-1.98	JAK-STAT signaling pathway
	$+1.14$	Calcium signaling pathway	-1.91	Apoptosis
			-1.75	Wnt signaling pathway
			-1.32	Cell cycle
$S1 \rightarrow C1\|M$	$+1.85$	**RET**	-3.52	Wnt signaling pathway
	$+1.19$	PPAR signaling pathway	-3.38	JAK-STAT signaling pathway
			-2.78	**PI3K-Akt signaling pathway**
			-2.76	Hedgehog signaling pathway
			-2.68	Notch signaling pathway
$S1 \rightarrow C4\|M$	$+4.20$	Calcium signaling pathway	-3.18	p53 signaling pathway
	$+3.89$	cAMP signaling pathway	-2.65	Cell cycle
	$+3.40$	MAPK signaling pathway	-1.99	Homologous recombination
	$+2.76$	Hedgehog signaling pathway	-1.64	Cytokine-cytokine receptor interaction
	$+2.72$	ECM-receptor interaction	-1.61	Apoptosis
$S2 \rightarrow C5$	$+3.67$	Cell cycle	-2.69	**RET**
	$+2.76$	Homologous recombination	-2.08	MAPK signaling pathway
	$+2.56$	p53 signaling pathway	-1.59	PPAR signaling pathway
	$+1.85$	mTOR signaling pathway	-1.43	cAMP signaling pathway
	$+1.79$	Adherens junction	-1.02	Hedgehog signaling pathway

Table S4. Perturbed pathways during the evolution of cell communities in primary and metastatic tumors (Case 4). The top five perturbed pathways whose gain or loss greater than 1.0 along each edge of phylogeny are shown.

Trajectory	Gain	Perturbed Pathways	Loss	Perturbed Pathways
$C3\|P \rightarrow S1$	$+2.38$	Homologous recombination	-4.49	Cytokine-cytokine receptor interaction
	$+1.56$	**ErbB signaling pathway**	-4.23	**PI3K-Akt signaling pathway**
	$+1.54$	Cell cycle	-4.10	JAK-STAT signaling pathway
	$+1.41$	cAMP signaling pathway	-3.97	Hedgehog signaling pathway
			-3.74	Apoptosis
$S1 \rightarrow S2$	$+1.89$	cAMP signaling pathway	-1.66	Notch signaling pathway
	$+1.69$	**ErbB signaling pathway**	-1.27	JAK-STAT signaling pathway
	$+1.47$	HIF-1 signaling pathway	-1.14	Apoptosis
	$+1.47$	ECM-receptor interaction	-1.01	Cytokine-cytokine receptor interaction
	$+1.43$	Calcium signaling pathway		
$S2 \rightarrow C1\|M$	$+1.43$	PPAR signaling pathway	-2.53	Notch signaling pathway
	$+1.19$	**RET**	-2.44	Wnt signaling pathway
	$+1.09$	p53 signaling pathway	-2.35	Hedgehog signaling pathway
			-2.32	JAK-STAT signaling pathway
			-1.66	VEGF signaling pathway
$S2 \rightarrow C4\|M$	$+4.40$	Calcium signaling pathway	-2.37	p53 signaling pathway
	$+3.91$	cAMP signaling pathway	-1.93	Cell cycle
	$+3.81$	ECM-receptor interaction	-1.74	Homologous recombination
	$+3.64$	MAPK signaling pathway		
	$+3.62$	Focal adhesion		
$S1 \rightarrow C2\|M$	$+1.84$	Homologous recombination	-3.07	TGF-beta signaling pathway
	$+1.39$	Cell cycle	-2.77	**PI3K-Akt signaling pathway**
			-2.69	ECM-receptor interaction
			-2.59	Focal adhesion
			-2.58	PPAR signaling pathway
